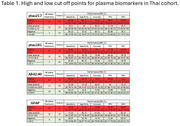# Validation of blood biomarkers for Alzheimer Disease in Thai Cohort

**DOI:** 10.1002/alz70856_102473

**Published:** 2025-12-25

**Authors:** Vorapun Senanarong, Tanyaluck Thientunyakit, Chatchawan Rattanabannakit, Natthamon Wongkom, Pathitta Dujada, Atthapon Raksthaput, Sunisa Chaichanettee, Paphawadee Phoyoo, Lertchai Wachirutmangur, Philip Scheltens

**Affiliations:** ^1^ Division of Neurology, Department of Medicine, Faculty of Medicine, Siriraj hospital, Mahidol university, Bangkok, Thailand; ^2^ Faculty of Medicine Siriraj Hospital, Mahidol University, Bangkok, Thailand; ^3^ Department of Radiology, Faculty of Medicine, Siriraj hospital, Mahidol University, Bangkok, Thailand; ^4^ Department of Medicine, Faculty of Medicine Siriraj Hospital, Mahidol University, Bangkok, Bangkok, Thailand; ^5^ Division of Neurology, Department of Medicine, Faculty of Medicine Siriraj Hospital, Mahidol University, Bangkok, Thailand; ^6^ Amsterdam Neuroscience, Neurodegeneration, Amsterdam, Netherlands; ^7^ EQT Life Sciences Partners, Amsterdam, 1071 DV Amsterdam, Netherlands; ^8^ Alzheimer Center Amsterdam, Neurology, Vrije Universiteit Amsterdam, Amsterdam UMC location VUmc, Amsterdam, Netherlands

## Abstract

**Background:**

Our objective of this study is to identify suitable local cut‐off values of plasma‐based biomarkers in Alzheimer disease (AD) in the discrimination of AD and non‐AD subgroups in Thai population.

**Method:**

We conducted a prospective study in 51 individuals with clinically diagnosed AD (15 had amyloid PET positive (≥ 30 centiloid, CL), 16 had CSF Aβ_42_ ≤547 pg/mL and CSF pTau 181 >57 pg/mL regarded as amyloid positive AD), 34 individuals with clinically diagnosed non‐AD dementia (9 had amyloid PET negative, 25 had CSF Aβ_42_ and CSF pTau181 negative), and 12 individuals with mild cognitive impairment (MCI) or normal controls (6 had amyloid PET negative and 6 had CSF Aβ_42_ and CSF pTau181 negative). Aβ‐PET using 18F‐florbetapir and plasma biomarkers (Aβ_40_, Aβ_42_, *p*‐tau181, pTau 217, Glial fibrillary acidic protein (GFAP)) were obtained in all subjects within 6 months before or after the PET study. The quantitative analysis of Aβ‐PET to obtain Centiloid (CL) followed the standard method using the SPM8 pipeline. Blood biomarker analysis utilized Simoa® Quanterix immunoassay. CSF Aβ42, t‐Tau and *p*‐Tau were measured separately, in duplicate, by commercially available sandwich ELISA kits (Innotest; Innogenetics/Fujirebio, Ghent, Belgium). Participants underwent a standardized diagnostic dementia evaluation.

**Result:**

Among 97 individuals, 54 (55.7%) amyloid positive and 43(44.3%) amyloid negative individuals were included in this blood biomarkers validation study. Mean age was 65.86(8.01) years, mean TMSE was 19.57(7.44) and mean MOCA was 15.48(7.27). Mean(SD) plasma Aβ 42/40, pTau181, pTau 217, NFL, and GFAP were 0.054(0.013) pg/mL, 37.591(18.975) pg/mL, 1.088(0.949) pg/mL, 102.703(412.308) pg/mL, and 162.459(107.223) pg/mL respectively. Utilizing single cut off from ROC analysis: the cut off points are as followed; for plasma Aβ42/40≤0.054pg/mL (AUC(SE)=0.716(0.064), *p* <0.0001, accuracy 72.63(62.52‐81.28)); plasma pTau181>32.7pg/mL (AUC(SE)=0.812(0.058), *p* <0.0001, accuracy73.20(63.24‐81.68)); plasma pTau217>0.522pg/mL (AUC(SE)=0.856(0.056), *p* <0.0001, accuracy87.14(76.99‐93.95)); GFAP>119pg/mL (AUC(SE)=0.796(0.058), *p* <0.0001, accuracy77.89(68.22‐85.77)). For high and low cut‐ points, the results were shown in table 1.

**Conclusion:**

Plasma biomarkers showed promising diagnostic performances and potential clinical usefulness in diagnosing dementia. Plasma *p*‐tau217 was best differentiates AD positive from AD negative groups, followed by plasma GFAP, pTau181, and Aβ42/40 ratio.

Thank you Health Systems Research Institute (Thailand) grant support for this study